# Multimodal dynamic hierarchical clustering model for post-stroke cognitive impairment prediction

**DOI:** 10.1186/s42492-025-00202-0

**Published:** 2025-09-01

**Authors:** Chen Bai, Tan Li, Yanyan Zheng, Gang Yuan, Jian Zheng, Hui Zhao

**Affiliations:** 1 Neurology Department, Wenzhou Third Clinical Institute Affiliated to Wenzhou Medical University, Wenzhou People’s Hospital, Wenzhou, Zhejiang 32500 China; 2 Department of Medical Imaging, Suzhou Institute of Biomedical Engineering and Technology, Chinese Academy of Sciences, Suzhou , Jiangsu 215163 China; 3 Neurology Department, The First Affiliated Hospital of Soochow University, Soochow University, Suzhou, 215006 Jiangsu China

**Keywords:** Post-stroke cognitive impairment, Multimodal neuroimaging, Graph neural network, Brain connectome, Hierarchical fusion

## Abstract

Post-stroke cognitive impairment (PSCI) is a common and debilitating consequence of stroke that often arises from complex interactions between diverse brain alterations. The accurate early prediction of PSCI is critical for guiding personalized interventions. However, existing methods often struggle to capture complex structural disruptions and integrate multimodal information effectively. This study proposes the multimodal dynamic hierarchical clustering network (MDHCNet), a graph neural network designed for accurate and interpretable PSCI prediction. MDHCNet constructs brain graphs from diffusion-weighted imaging, magnetic resonance angiography, and T1- and T2-weighted images and integrates them with clinical features using a hierarchical cross-modal fusion module. Experimental results using a real-world stroke cohort demonstrated that MDHCNet consistently outperformed deep learning baselines. Ablation studies validated the benefits of multimodal fusion, while saliency-based interpretation highlighted discriminative brain regions associated with cognitive decline. These findings suggest that MDHCNet is an effective and explainable tool for early PSCI prediction, with the potential to support individualized clinical decision-making in stroke rehabilitation.

**Supplementary Information:**

The online version contains supplementary material available at 10.1186/s42492-025-00202-0.

## Introduction

Post-stroke cognitive impairment (PSCI) affects approximately 30%–50% of stroke survivors and significantly impairs long-term recovery [1, 2]. The neuropathology underlying PSCI is highly heterogeneous and involves focal lesions and distributed network disruptions, which complicates early diagnosis and individualized treatment planning [3, 4]. Given this complexity, the development of effective models capable of capturing the heterogeneity of brain networks has become critically important.

Clinically, machine learning-based neuropsychological scales and clinical assessments represent the most widely adopted tools for PSCI evaluation. For example, Kandiah et al. [5] and Lee et al. [6] demonstrated the effectiveness of standardized cognitive scales and clinical data in predictive models. With advancements in magnetic resonance imaging (MRI) technology, neuroimaging-based machine learning methods have gained prominence. Recent studies [7, 8] have successfully implemented conventional machine learning algorithms, including logistic regression and support vector machines (SVM), for PCSI prediction using imaging data. Although machine learning has achieved superior performance in predicting PSCI, these methods are fundamentally limited by their reliance on handcrafted features and shallow learning architectures, which may not capture the complex nonlinear relationships inherent in brain network dynamics. This limitation becomes particularly pronounced when dealing with the heterogeneous and high-dimensional nature of poststroke brain reorganization, thus motivating the exploration of more sophisticated computational approaches.

Deep learning has advanced medical image analysis; however, convolutional neural networks exhibit intrinsic limitations in modeling nonlocal brain network dynamics [9]. Their Euclidean inductive bias fails to capture critical spatially disconnected lesions, which can have network-level effects despite being anatomically disconnected. Graph convolutional networks (GCNs) [10, 11] offer a promising alternative to modeling topological relationships within brain networks. For example, Iporre-Rivas et al. [12] used GCN-based activation mapping to visualize stroke-induced functional compensation patterns. However, most existing GCN-based approaches [13–15] are restricted to unimodal neuroimaging inputs and often neglect critical clinical variables and cross-modal interactions. An effective PSCI prediction model should integrate diverse imaging modalities and clinical information in a biologically meaningful manner.

Moreover, structural brain networks present an inherent redundancy that constrains effective model design [4, 16]. Specifically, not all brain regions contribute equally to the pathological process of PSCI, as many nodes may have minimal or no relevance to cognitive impairment. This creates computational redundancy that reduces the model’s efficiency and potentially compromises the downstream classification performance. Standard graph pooling methods attempt to address this issue but often fail to consider the hierarchical characteristics of brain networks, leading to the loss of critical topological structures [11, 17–19]. For instance, Chen et al. [20] achieved 22% acceleration in model convergence through aggressive feature compression. However, it simultaneously causes degradation of hippocampal-prefrontal tract integrity, which is a known biomarker of memory consolidation that is crucial for PSCI assessment. This is particularly problematic in the context of PSCI, in which lesion-induced network reorganization occurs at multiple scales, including local circuits, long-range hubs, and global efficiency. To address these trade-offs, this study draws upon neuropathological evidence suggesting stratified network degeneration, where lesion-induced excitotoxicity disrupts local microcircuits, secondary diaschisis affects hub regions, and tertiary reorganization reduces global efficiency. These insights motivated the design of a multimodal dynamic hierarchical clustering neural network (MDHCNet) that performs selective redundancy reduction using lesion-aware hierarchical clustering (HC) while preserving essential hub connectivity.

To tackle these challenges, this study proposes the MDHCNet. MDHCNet enhances structural and functional brain network modeling through three key components. First, a node enhancement module adaptively fuses regional radiomic features with clinical variables via attention-based weighting to establish clinically meaningful associations between brain network nodes and PSCI risk. Subsequently, a dynamic edge enhancement module integrates brain network damage data, anatomical adjacency information, and multihead attention mechanisms to model both acute and chronic connectivity changes across the post-stroke phases. Then, a HC module implements topology-aware dimensionality reduction guided by a custom clustering loss function, selectively preserving the critical connections identified through graph theoretical analysis. Finally, the resulting feature representation is processed through fully connected layers for the final classification, striking a balance between sparsity and topological fidelity. Extensive evaluations on a real-world stroke cohort demonstrate the superior predictive performance and interpretability of MDHCNet for early PSCI detection. In our previous study [21], we found that integrating brain network damage information significantly improved the prediction of PSCI, providing a foundation for the multimodal data integration in this study. In addition, this work introduces GCNs and a HC mechanism to better capture the topological relationships between brain regions and enhance interpretability, particularly in identifying clinically meaningful brain areas.

The main contributions of this study are as follows:

1. A multimodal brain network framework is developed to integrate clinical data with radiomic and brain network damage features, leveraging attention-guided enhancements for both node and edge representations.
2. A lesion-aware HC module is introduced to reduce graph redundancy while preserving essential topological structures, particularly functionally critical hub regions.
3. Extensive experiments demonstrate the superior predictive performance and interpretability of MDHCNet in modeling post-stroke network reorganization.

**Fig. 1:**
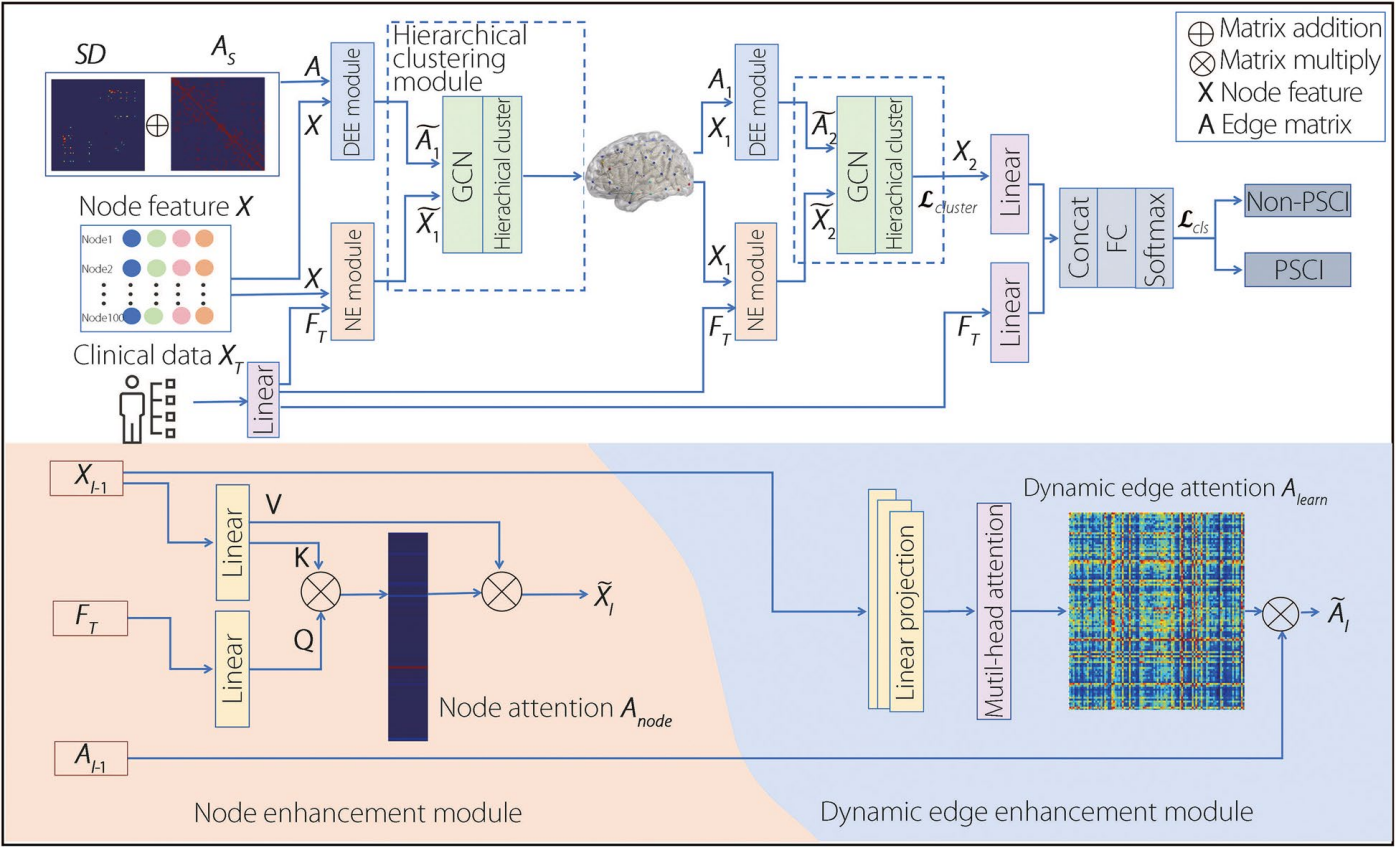
Overview of proposed model framework. First, a node enhancement module is constructed by integrating regional radiomic features with PSCI-relevant clinical variables. Next, a dynamic edge enhancement module models edge connections using lesion-derived brain network damage information, anatomical adjacency, and multi-head attention mechanisms. Then, a HC module with a custom clustering loss is employed for topology-aware dimensionality reduction, preserving critical connectomic structures. Finally, a fully connected layer performs classification for PSCI prediction

## Methods

### Overall framework

MDHCNet addresses the limitations of brain network redundancy and insufficient multimodal integration through three optimization stages (Fig. 1). First, the node enhancement module integrates radiomic features and PSCI-related clinical data via an adaptive graph attention mechanism. Second, dynamic edge modules incorporate brain network damage information, anatomical adjacency, and multihead attention to capture connectivity alterations across post-stroke phases. Third, HC reduces redundancy while preserving the critical topological features. Finally, a fully connected layer performs classification based on refined graph representations.

**Fig. 2:**
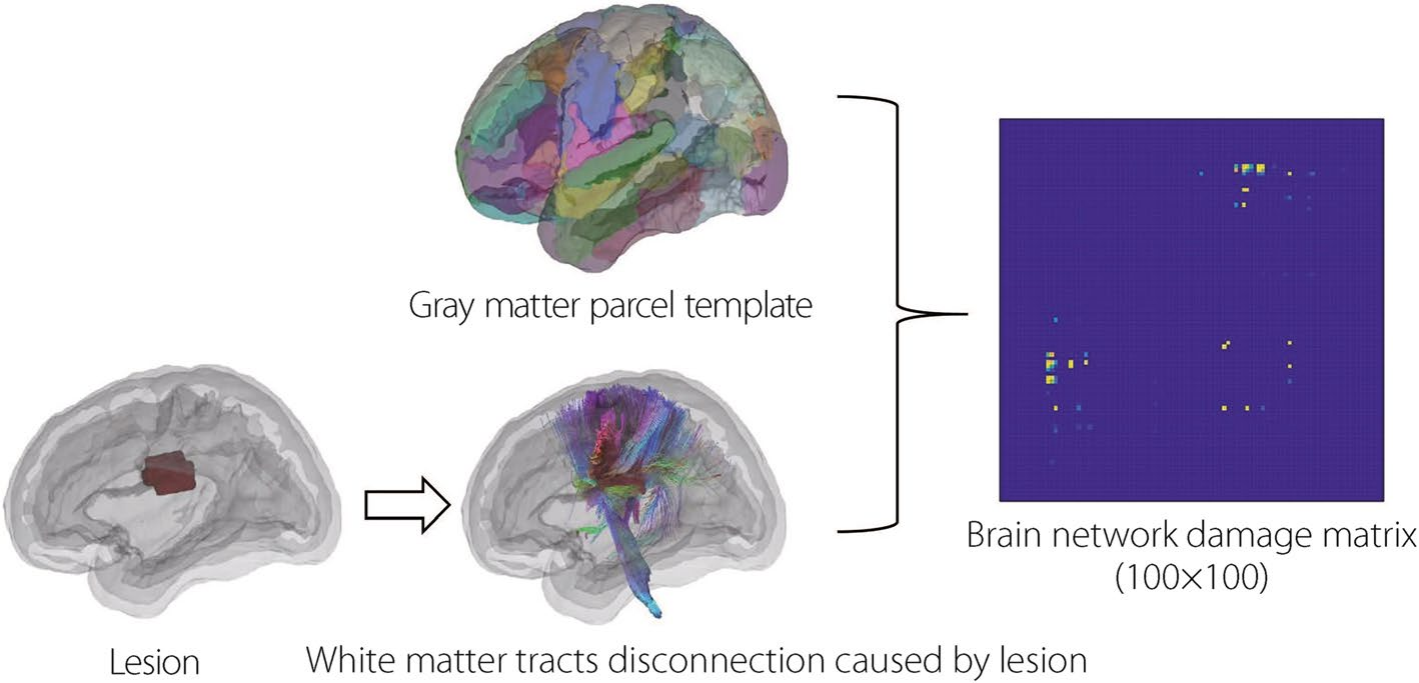
Schematic of brain network damage matrix construction. Each patient’s lesion mask was embedded into a standardized white matter tractography atlas to assess disconnections. Based on the Schaefer-Yeo parcellation of cortical gray matter, the number of disrupted streamlines between parcel pairs was quantified. The density of disconnected streamlines was computed and converted into a percentage matrix, representing the severity of regional disconnections and forming the individualized brain network damage matrix

**Fig. 3:**
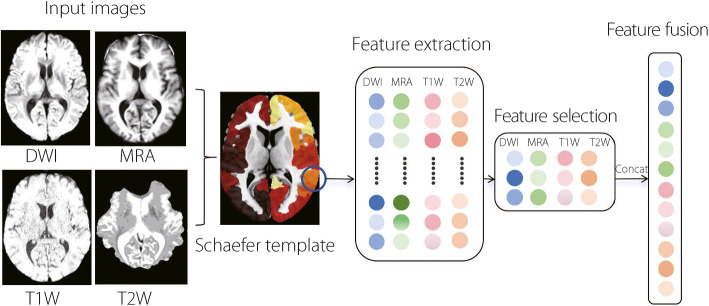
Illustration of node feature extraction for each brain region. From each brain region defined by the Schaefer-Yeo atlas, 851 radiomic features were extracted across four MRI modalities. For each region-modality pair, 30 informative features were selected using Spearman correlation and the minimum redundancy maximum relevance algorithm. The selected features from the four modalities were concatenated to form a multimodal node feature vector for each brain region

### Preparation of brain network features

The preprocessing pipeline established two complementary representations of post-stroke network pathology: (1) lesion-induced brain network SD patterns and (2) multimodal nodal signatures reflecting regional microstructural alterations. These components collectively provide a foundation for the subsequent graph-based analyses.

#### Construction of brain network damage matrix

To quantify structural damage (SD), a brain network damage matrix was constructed by integrating lesion quantitative tractography [3] data with the Schaefer-Yeo cortical parcellation Atlas [22]. The procedure comprised three steps: (1) A standardized structural connectivity matrix was established using a tractogram-based connectome and Schaefer-Yeo parcellation (registered to the MNI152 space), where the edges represent normative streamline counts between gray matter parcels. (2) Each patient’s lesion mask was overlaid onto normative tractography to identify the disrupted white matter tracts. The number of streamlines that terminated prematurely within the same pair of parcels was counted to obtain a parcel-wise disconnection density matrix. (3) This matrix was normalized by the total number of normative streamlines to derive a disconnection severity matrix, which served as the brain network damage matrix used in the subsequent modeling (Fig. 2).

**Table 1:** Selected radiomics features across MRI sequences

Radiomics feature	Total	DWI	MRA	TIW	T2W
Shape features	14	-	-	-	-
First-order features	18		2	1	1
GLCM	24	-	-	-	-
GLRLM	16	-	-	-	-
GLSZM	16	2	1	1	1
GLDM	14	-	-	-	-
NGTDM	5	-	-	-	-
Wavelet-HHH	93	6	4	5	4
Wavelet-HHL	93	5	4	4	5
Wavelet-HLH	93	3	4	5	4
Wavelet-HLL	93	3	2	3	3
Wavelet-LHH	93	4	4	5	3
Wavelet-LHL	93	3	3	2	3
Wavelet-LLH	93	3	4	2	4
Wavelet-LLL	93	1	2	2	2
**Total**	851	30	30	30	30

Note: The 'Total' column represents the total number of radiomics features that can be extracted for each feature category from a single modality, while the numbers under each modality column (DWI, MRA, T1W, T2W) represent the number of features selected after feature selection for that specific modality. *L* Low-pass filter, *H* High-pass filter. HHH, HHL, etc., denote 3D wavelet decomposition directions. *GLCM* Gray-level co-occurrence matrix, *GLRLM* Gray-level run-length matrix, *GLSZM* Gray-level size zone matrix, *GLDM* Gray-level dependence matrix, *NGTDM* Neighboring gray-tone difference matrix

#### Construction of brain network mapping

A cortical brain graph was constructed, and representative node features were extracted for downstream multimodal analysis, as shown in Fig. 3. The steps were as follows: (1) Graph construction: A cortical graph was defined using the Schaefer-Yeo parcellation with 100 cortical regions serving as nodes. Undirected edges were initialized based on anatomical adjacency; that is, if two cortical regions were directly adjacent, an edge was created between the corresponding nodes; otherwise, no edge was included. Subsequently, the parcellation was registered in the MNI152 standard space. (2) Node feature extraction: For each of the four MRI modalities (diffusion-weighted imaging (DWI), magnetic resonance angiography (MRA), T1-weighted imaging (T1W), and T2-weighted imaging (T2W)), 851 radiomic features were extracted per region using the PyRadiomics library [23]. To remove redundancies and retain cognitively relevant features, a two-step selection process was employed. First, Spearman correlation analysis (threshold = 0.95) was applied to each region to eliminate highly correlated features. Second, the minimum redundancy maximum relevance (mRMR) method [24] was used to select 100 informative and relatively independent features per modality per region. Next, the features were ranked based on their frequency across all 100 regions within each modality, and the top 30% of the most frequently selected features were retained. Finally, 120 features per node were obtained by aggregating features across all four modalities. The details of the feature selection for each modality are presented in Table 1.

### Node enhancement module

To enhance the representation of brain network nodes, a node-enhancement module that integrates clinical information relevant to PSCI was proposed. This module leverages an attention mechanism to adaptively capture the relationship between clinical features and brain network topology, thereby improving the model’s predictive ability for PSCI.

First, the original clinical features $X_{t}\in \mathbb {R}^{1\times D_t}$ are transformed through a linear layer using LeakyReLU [25] activation, as follows:

1 $$\begin{aligned} F_t = LReLU(X_t W_t+b_t) \end{aligned}$$

where $W_t\in {\mathbb {R}^{D_t\times D_h}}$ is the linear layer weight matrix, $b_t\in {\mathbb {R}^{D_h}}$ is the bias term, and $D_h$ denotes the hidden dimension aligned with the node feature space. Next, an attention mechanism [26] is used to model the interaction between the transformed clinical features $F_t$ and node features $X\in {\mathbb {R}^{N\times D_h}}$, where *N* is the number of brain regions. The attention weights were computed as follows:

2 $$\begin{aligned} Q=W_Q F_t, K=W_KX, V=W_VX \end{aligned}$$

3 $$\begin{aligned} A_{node}=softmax\left(\frac{QK^T}{\sqrt{D_k}}\right) \in \mathbb {R}^{1\times N} \end{aligned}$$

where *Q*, *K*, *V* are the query, key, and value vectors, respectively. $D_k$ denotes the dimensions of the key vector. The resulting attention vector $A_{node}$ reflects the importance of each brain region with respect to the cognitive impairment prediction. Finally, the node features are updated using the attention-weighted sum of the value vectors.

4 $$\begin{aligned} \tilde{X}_l=A_{node}V \in \mathbb {R}^{N\times D_h} \end{aligned}$$

This mechanism enables the model to emphasize brain regions that are clinically relevant to PSCI while suppressing irrelevant regions, thereby enhancing the interpretability and diagnostic accuracy (ACC) of the model.

### Dynamic edge module

To model post-stroke alterations in structural brain connectivity, a dynamic edge module was proposed to adaptively refine graph topology using both anatomical priors and lesion-derived damage information. Specifically, the initial edge prior was constructed by integrating two sources: a SD matrix $SD \in \mathbb {R}^{N \times N}$, which quantifies the connectivity loss between brain regions owing to stroke, and an anatomically plausible adjacency matrix $A_s \in \mathbb {R}^{N \times N}$. The fused edge prior is defined as

5 $$\begin{aligned} A_{prior} = SD + A_s \end{aligned}$$

This formulation preserves the core anatomical topology while incorporating individualized pathological disruptions.

To dynamically update the connectivity pattern during learning, a multihead self-attention mechanism was applied over the node features $X$. For each attention head $h \in \{1, \dots , H\}$, the node features are projected linearly onto queries $Q^h \in \mathbb {R}^{N \times D_k}$, keys $K^h \in \mathbb {R}^{N \times D_k}$. The attention score for each head is calculated as follows:

6 $$\begin{aligned} A_{learn}^h = \text {softmax}\left( \frac{Q^h {K^h}^T}{\sqrt{D_k}}\right) \end{aligned}$$

The final learned adjacency matrix $A_{learn} \in \mathbb {R}^{N \times N}$ is obtained by averaging across all heads as follows:

7 $$\begin{aligned} A_{learn} = \frac{1}{H}\sum \limits _{h=1}^{H} A_{learn}^h \end{aligned}$$

This averaging strategy mitigates noise and stabilizes the attention-based topology refinement, yielding more interpretable and robust connectivity estimates.

At each layer $l$, the refined adjacency matrix is obtained by element-wise multiplication between the learned attention-based connectivity and the prior edge matrix from the previous layer:

8 $$\begin{aligned} \tilde{A}_l =A_{learn} A_{l-1} \end{aligned}$$

For the first layer ($l = 1$), $A_0 = A_{prior}$, which are the combined anatomical and pathological priors. For subsequent layers, $A_{l-1}$ is the output from the previous dynamic edge module, which enables progressive topology refinement across the layers.

### HC module

To effectively capture the essential topological features of brain networks while reducing the model complexity, a HC module was designed as a core component of the GCN architecture. This module performs node aggregation based on feature similarity and structural connectivity, thereby enabling dimensionality reduction and enhancing generalization. This is particularly valuable for revealing the neural mechanisms underlying PSCI.

After each GCN layer, an HC is inserted to perform node aggregation. The normalized adjacency matrix is computed using $\hat{D}_{ii}=\sum _{j=0}A_{ij}$, and the graph convolution is formulated as

9 $$\begin{aligned} GCN(\tilde{X}) = \hat{D}^{-1/2} \tilde{A} \hat{D}^{-1/2} \tilde{X} W \end{aligned}$$

Given the node feature matrix $\tilde{X}\in \mathbb {R}^{N\times D}$ and adjacency matrix $\tilde{A}\in \mathbb {R}^{N\times N}$ obtained after GCN processing, the HC module proceeds as follows:

**Step 1: Distance matrix construction.** Two types of pairwise distances were computed to integrate topological and feature information. The structural distance $D_{path}$ captures the shortest path length between nodes *i* and *j*, derived from the adjacency matrix *A*:

10 $$\begin{aligned} D_{path}(i,j) = ShortestPath(\tilde{A},i,j) \end{aligned}$$

The feature-based distance $D_{features}$ is defined as the squared Euclidean distance between node embeddings:

11 $$\begin{aligned} D_{features}(i,j) = \Vert \tilde{X}_i-\tilde{X}_j\Vert _2^2 \end{aligned}$$

Finally, these two distance matrices were combined into a unified distance matrix $D_{combined}$ via weighted fusion. The weight coefficient $\alpha$ balances the contributions of both distances, set to 0.5, to emphasize both the network topology and node feature information equally:

12 $$\begin{aligned} D_{combined} = \alpha D_{path} + (1-\alpha )D_{features} \end{aligned}$$

**Step 2: Clustering assignment matrix construction.** HC was performed using a linkage method based on a combined distance matrix. The clustering result *Z* is a dendrogram that records the order and distances of node merges.

13 $$\begin{aligned} Z = linkage(D_{combined}) \end{aligned}$$

Here, *Z* represents a HC tree that details the merging sequence and distances. Based on the desired number of clusters *C*, *N* nodes are grouped into *C* clusters, and a cluster label is assigned to each node. The labels are then transformed into a clustering assignment matrix $S \in \mathbb {R}^{N\times C}$.

**Step 3: Graph pooling operation.** Using the clustering assignment matrix *S*, a graph pooling operation was performed to obtain a reduced-dimensional representation of both the node features and the adjacency matrix. This step aggregates the original *N* nodes into *C* supernodes, where each supernode represents a node cluster.

14 $$\begin{aligned} X = S^T\tilde{X}, A = S^T\tilde{A}S \end{aligned}$$

After pooling, the node feature matrix is updated to $X \in \mathbb {R}^{C\times D}$ and the adjacency matrix is updated to $A \in \mathbb {R}^{C\times C}$.

### Classification module

After obtaining the compact feature matrix and corresponding adjacency matrix from the HC module, graph-level classification was performed to identify subjects with PSCI. A global average pooling operation was applied to the final node feature matrix $X \in \mathbb {R}^{C \times D}$ to generate a graph-representation vector.

15 $$\begin{aligned} g_f = concat(FC(F_T), FC(X)) \end{aligned}$$

This vector is then passed through a fully connected layer, followed by softmax activation to produce the predicted class probabilities:

16 $$\begin{aligned} \hat{y}=softmax(FC(g_f)) \end{aligned}$$

The model is trained using the cross-entropy loss between $\hat{y}$ and the true label *y*.

This classification module, in combination with the previous components, ensures that the model captures meaningful high-level graph representations while maintaining the ability to generalize across subjects. This completes the end-to-end architecture of the proposed network for PSCI identification.

### Loss function

To optimize both the classification performance and structural quality of the learned brain graphs, a multi-objective loss function composed of three components was adopted. First, classification loss ensures accurate discrimination between PSCI and non-PSCI subjects, formulated as cross-entropy:

17 $$\begin{aligned} L_{cls} = -\frac{1}{M}\sum \limits _{m=1}^{M}\sum \limits _{n=1}^{N} y_{m,n}log(p_{m,n}) \end{aligned}$$

where *M* is the number of samples, *N* is the number of classes, $y_{m,n}$ denotes the true label (0 or 1) of category *n* to which sample *m* belongs, and $p_{m,n}$ is the predicted probability.

Second, link prediction loss encourages the preservation of the graph topology during HC. Given the cluster assignment matrix $S \in \mathbb {R}^{C_{l-1}\times C_l}$, where $C_{l-1}$ denotes the number of nodes output from the previous layer and $C_{1}$ denotes the number of nodes in the current clustered graph, the adjacency matrix from the previous layer is $A\in \mathbb {R}^{C_{l-1}\times C_{l-1}}$. The reconstruction error is formulated as

18 $$L_\text{link}=\left\|A-SS^\text{T}\right\|_F^2$$

where $\Vert{\cdot}\Vert_F$ denotes the Frobenius norm, which corresponds to the square root of the sum of the squared entries of a matrix. Finally, the entropy loss regularizes the clustering assignment by promoting confident (i.e., low-entropy) cluster membership: 

19 $$\begin{aligned} L_{entropy}=-\sum S log(S) \end{aligned}$$

The total objective is the sum of all three losses.

20 $$\begin{aligned} L_{total}= L_{cls}+L_{link}+L_{entropy} \end{aligned}$$

## Data profile

Data from 163 patients diagnosed with lacunar infarction via neuroimaging were retrospectively collected at the collaborating hospital between April 2018 and May 2024. Lacunar infarctions were defined as acute ischemic lesions with a diameter $\le$ 20 mm located in the perforating artery territories on DWI following standard criteria [27].

After quality control, patients were excluded due to motion artifacts or excessive lesion burden ($n=4$), or incomplete MRI sequences ($n=7$). Ultimately, 152 patients with complete baseline and follow-up data were included in the analysis. Based on the montreal cognitive assessment (MoCA) scores at the 3-month follow-up, 44 patients were classified as having PSCI and 108 as having non-PSCI.

## Clinical data collection

Demographic and clinical data were collected within 48 hours of admission, including sex, age, cerebral microbleeds, deep white matter hyperintensities, total burden scores, perivascular space grading (grades 2–4), the presence of microbleeds, and national institutes of health stroke scale (NIHSS) scores. A full summary of the baseline demographic and clinical characteristics of the PSCI and non-PSCI groups is shown in Table 2.

Cognitive status was assessed three months post-stroke using the MoCA administered by trained neurologists during outpatient follow-up visits. Cognitive impairment was determined using the Chinese-revised protocol from the Vascular Impairment of Cognition Classification Consensus Study: a MoCA score $\le$ 13 for illiterate patients, $\le$ 19 for those with primary education, and $\le$ 24 for those with junior high school education or higher.

**Table 2:** Baseline characteristics of study participants

Characteristics	Non-PSCI	PSCI	*P* value
Number	108	44	-
Age (years)	64.05 ± 10.49	68.43 ± 9.58	0.018
Gender (female/male)	39/69	18/26	0.579
Female	39 (36.1%)	18 (40.9%)	-
Male	69 (63.9%)	26 (59.1%)	-
Microbleed (no/yes)	86/22	30/14	0.132
No	86 (79.6%)	30 (68.2%)	-
Yes	22 (20.4%)	14 (31.8%)	-
PVS (0–1/2–4 grade)	59/49	16/28	0.041
0–1 grade	59 (54.6%)	16 (36.4%)	-
2–4 grade	49 (45.4%)	28 (63.6%)	-
Periventricular hyperintensity	1.13 ± 1.11	2.11 ± 0.95	< 0.001*
Deep hyperintensity	0.67 ± 0.84	1.57 ± 1.00	< 0.001*
Total burden score	1.68 ± 1.08	2.50 ± 1.05	< 0.001*
NIHSS	2.33 ± 2.43	3.77 ± 3.06	0.003

Note: Continuous variables are presented as mean ± SD; categorical variables as number (%). * Significant variables between PSCI and non-PSCI ($P<0.001$)

## Imaging acquisition and preprocessing

Four MRI sequences were acquired: DWI, MRA, T1W, and T2W images. Lesions were manually segmented on DWI images by two experienced radiologists using ITK-SNAP (http://www.itksnap.org) and subsequently reviewed and corrected by a senior neurologist.

All modalities underwent the following preprocessing steps: (1) motion correction and bias field correction were applied to reduce motion-related artifacts and signal nonuniformities, (2) the N3 algorithm was used to correct intensity inhomogeneity, (3) Talairach transformation was applied to align individual images into a standardized stereotactic space, (4) intensity normalization was performed to mitigate inter-subject and scanner-related signal variations, and (5) skull stripping using FreeSurfer.

For T1W images, additional preprocessing steps included (1) spatial normalization to the MNI152 template (1 $\tt mm^3$ resolution) using SPM12 (https://www.fil.ion.ucl.ac.uk/spm/). The preprocessed DWI, MRA, T2W images, and corresponding lesion masks were then co-registered to the normalized T1W image using SPM12. All modalities were resampled to a uniform spatial resolution of $182\times 218\times 182$ voxels.

## Results

### Optimization details

Five-fold cross-validation was conducted, with 80% of the data used for training and 20% for validation. Stratified sampling ensured balanced proportions of PSCI and non-PSCI patients across the folds. Model performance was evaluated using four metrics: ACC, area under the ROC curve (AUC), sensitivity (SEN), and specificity (SPE).

The model was trained for 500 epochs using the Adam optimizer, with a learning rate of 1e-5 and momentum coefficients ranging from 0.9 to 0.999. A step-learning-rate scheduler (step size = 20) was applied. The loss function was composed of binary cross-entropy with auxiliary terms, and the final predictions were obtained using softmax. To reduce overfitting, dropout ($P=0.5$) was applied before the final fully connected layer. Additionally, data augmentation was performed using random noise injection and affine transformations.

All experiments were implemented in Python 3.9 using PyTorch 1.13 (https://pytorch.org) and executed on an NVIDIA GeForce RTX 3090Ti GPU.

**Table 3:** Ablation study of node and edge enhancement modules

$A_{node}$	$A_{learn}$	SD	ACC	AUC	SEN	SPE
$\times$	$\checkmark$	$\checkmark$	0.824 ± 0.026	0.791 ± 0.063	0.705 ± 0.100	0.875 ± 0.044
$\checkmark$	$\times$	$\checkmark$	0.793 ± 0.047	0.739 ± 0.101	0.651 ± 0.253	0.865 ± 0.060
$\checkmark$	$\checkmark$	$\times$	0.824 ± 0.026	0.745 ± 0.078	0.614 ± 0.092	**0.906 ± 0.027**
$\checkmark$	$\times$	$\times$	0.802 ± 0.034	0.776 ± 0.079	0.676 ± 0.055	0.855 ± 0.041
$\checkmark$	$\checkmark$	$\checkmark$	**0.851 ± 0.039**	**0.795 ± 0.097**	**0.755 ± 0.167**	0.893 ± 0.043

**Table 4:** Ablation study of HC module

Method	Cluster	$L_{link}$	$L_{entropy}$	ACC	AUC	SEN	SPE
Baseline	$\times$	$\times$	$\times$	0.804 ± 0.040	0.795 ± 0.104	0.680 ± 0.183	0.856 ± 0.072
w/o Loss	$\checkmark$	$\times$	$\times$	0.793 ± 0.039	0.709 ± 0.125	0.710 ± 0.212	0.839 ± 0.063
w/$\mathcal {L}_{\text {link}}$	$\checkmark$	$\checkmark$	$\times$	0.829 ± 0.047	**0.847 ± 0.062**	**0.785 ± 0.111**	0.847 ± 0.066
w/$\mathcal {L}_{\text {Entropy}}$	$\checkmark$	$\times$	$\checkmark$	0.790 ± 0.037	0.733 ± 0.147	0.671 ± 0.162	0.839 ± 0.018
MDHCNet	$\checkmark$	$\checkmark$	$\checkmark$	**0.851 ± 0.039**	0.795 ± 0.097	0.755 ± 0.167	**0.893 ± 0.043**

### Ablation studies

To evaluate the effectiveness of each key component in MDHCNet, a series of ablation studies were conducted, focusing on three modules: node enhancement, dynamic edge enhancement, and HC.

**Node enhancement.** To assess the contribution of the clinical data-guided node enhancement module, a variant of MDHCNet was constructed without this component. As shown in Table 3, removing the node enhancement resulted in a decline in all classification metrics. Specifically, the ACC dropped to $0.824 \pm 0.026$, AUC to $0.791 \pm 0.063$, SEN to $0.705 \pm 0.100$, and SPE to $0.875 \pm 0.044$. These results demonstrate that incorporating clinical information into node representation significantly improves the overall model performance.

**Dynamic edge enhancement.** This module comprises two critical components: dynamic edge learning ($A_{learn}$) and SD features. To investigate their contributions, three ablation settings were tested: (1) removing $A_{learn}$, (2) excluding SD information, and (3) retaining only static edges while including both $A_{learn}$ and SD. As shown in Table 3, omitting either component leads to a performance degradation. For example, without $A_{learn}$, ACC decreased to $0.793 \pm 0.047$ and SEN dropped sharply to $0.651 \pm 0.253$. Without SD features, ACC was $0.824 \pm 0.026$, but SEN decreased to $0.614 \pm 0.092$. These results confirm that both components are essential for effectively capturing structural and functional disruptions in the brain network.

**HC.** To examine the impact of the HC module, four ablation experiments were conducted: (1) excluding the module entirely (i.e., no clustering, no $\mathcal {L}_{\text {link}}$, and no $\mathcal {L}_{\text {entropy}}$); (2) using only the clustering structure without loss terms; (3) adding only $\mathcal {L}_{\text {link}}$; and (4) adding only $\mathcal {L}_{\text {entropy}}$. As shown in Table 4, the absence of this module resulted in a decrease of approximately 5% in ACC, 7.5% in SEN, and 4% in SPE. Using only the clustering operation without loss optimization yielded an ACC of $0.793 \pm 0.039$, and all metrics underperformed compared with the complete model. When incorporating $\mathcal {L}_{\text {link}}$ alone, ACC improved to $0.829 \pm 0.047$, whereas using only $\mathcal {L}_{\text {entropy}}$ resulted in suboptimal results. Overall, the full clustering module with both loss terms achieved the best performance, highlighting its importance in enhancing model expressiveness and classification ACC.

**Table 5:** Comparison of graph pooling methods

Method	ACC	AUC	SEN	SPE
TopKPool [28]	0.797 ± 0.039	0.779 ± 0.091	0.846 ± 0.058	0.676 ± 0.137
KMeans [29]	0.782 ± 0.020	0.711 ± 0.076	0.671 ± 0.162	0.829 ± 0.052
Graclus [30]	0.795 ± 0.060	0.730 ± 0.121	0.853 ± 0.103	0.660 ± 0.070
DiffPool [31]	0.824 ± 0.026	**0.807** ± **0.037**	**0.864** ± **0.047**	0.723 ± 0.113
MDHCNet	**0.851** ± **0.039**	0.795 ± 0.097	0.755 ± 0.167	**0.893** ± **0.043**

### Influence of the graph pooling method

To validate the suitability and advantages of the proposed HC strategy, the method was compared to four widely used graph-pooling methods. (1) TopKPool [28], which selects top-scoring nodes to retain local information. (2) KMeans [29], which partitions nodes into clusters based on similarity. (3) Graclus [30], which adopts a greedy bottom-up strategy to merge nodes while preserving graph structure. (4) DiffPool [31], a state-of-the-art (SOTA) method that learns a soft assignment matrix for a hierarchical graph representation.

The proposed method integrates node features and graph structural information and jointly optimizes $\mathcal {L}_{\text {link}}$ and $\mathcal {L}_{\text {entropy}}$ to balance local detail retention and global structure preservation.

As shown in Table 5, the proposed method achieves an ACC of $0.851 \pm 0.039$, surpassing DiffPool ($0.824 \pm 0.026$) and significantly outperforming TopKPool, KMeans, and Graclus (all around 0.795). In terms of SPE, the proposed model yields $0.893 \pm 0.043$, far exceeding that of the other methods (DiffPool being the highest baseline at $0.723 \pm 0.113$), indicating an improved false-positive control. Furthermore, the model shows a superior SEN ($0.755 \pm 0.167$) compared to KMeans. These results demonstrate that the HC method captures the key features of brain networks more effectively while maintaining their hierarchical structure, thereby supporting more reliable clinical predictions.

**Table 6:** Comparison of clustering *C* values

*C*	ACC	AUC	SEN	SPE
8	0.770 ± 0.054	0.739 ± 0.102	0.676 ± 0.104	0.810 ± 0.057
16	**0.851** ± **0.039**	0.795 ± 0.097	0.755 ± 0.167	0.893 ± 0.043
24	0.824 ± 0.057	0.810 ± 0.096	0.688 ± 0.169	0.883 ± 0.047
32	0.804 ± 0.040	0.777 ± 0.084	0.775 ± 0.185	0.819 ± 0.038
40	0.783 ± 0.083	0.743 ± 0.159	0.726 ± 0.173	0.805 ± 0.068
48	0.787 ± 0.041	0.739 ± 0.049	0.726 ± 0.096	0.812 ± 0.055
56	0.838 ± 0.040	0.781 ± 0.082	0.635 ± 0.160	**0.924** ± **0.073**
64	0.787 ± 0.058	0.682 ± 0.115	0.636 ± 0.132	0.845 ± 0.046
72	0.806 ± 0.033	0.760 ± 0.060	0.561 ± 0.127	0.903 ± 0.079
80	0.806 ± 0.068	0.696 ± 0.115	0.643 ± 0.097	0.873 ± 0.061
88	0.804 ± 0.054	**0.830** ± **0.082**	**0.821** ± **0.066**	0.798 ± 0.078
96	0.779 ± 0.047	0.759 ± 0.061	0.656 ± 0.125	0.828 ± 0.027

### Influence of *C* in HC module

To determine the optimal number of clusters, *C*, experiments were conducted across twelve settings: $C \in \{8, 16, 24, 32, 40, 48, 56, 64, 72, 80, 88, 96\}$. In this framework, the number of clusters in the second layer was fixed at *C*/2.

As shown in Table 6, $C=16$ yields the best overall ACC ($0.851 \pm 0.039$). ACC exhibits a non-monotonic trend as *C* increases, suggesting that both under-clustering and over-clustering negatively impact performance. Notably, $C=56$ also achieves a competitive ACC of $0.838 \pm 0.040$. In terms of AUC, the highest value ($0.830 \pm 0.082$) is observed at $C=88$, indicating strong discriminative capability. However, its relatively lower ACC highlights the trade-off between precision and generalizability. For SEN, $C=88$ achieves the best score ($0.821 \pm 0.066$), whereas $C=56$ achieves the highest SPE ($0.924 \pm 0.073$). Overall, $C=16$ strikes the best balance across ACC, AUC ($0.795 \pm 0.097$), SEN ($0.755 \pm 0.167$), and SPE ($0.893 \pm 0.043$), making it the most robust configuration.

### Influence of different MRI data

To address the contribution of each imaging modality and validate the necessity of multimodal integration, ablation experiments were conducted using single-modality variants of MDHCNet. (1) MDHCNet-DWI: using only DWI features; (2) MDHCNet-MRA: using only MRA features; (3) MDHCNet-T1W: using only T1W features; (4) MDHCNet-T2W: using only T2W features; and (5) MDHCNet-all: using four MRI data (DWI, MRA, T1W, and T2W) features.

Table 7 shows that among single-modality models, MDHCNet-MRA achieved the best performance (ACC: 0.795 ± 0.055, AUC: 0.762 ± 0.099), while MDHCNet-T1W showed the lowest performance. Importantly, the complete MDHCNet-all model significantly outperformed all single-modality variants, achieving a 5.6% improvement in ACC and a 3.3% improvement in AUC compared to the best single-modality approach, demonstrating the effectiveness of multimodal integration.

**Table 7:** Comparison of different MRI data

Method	ACC	AUC	SEN	SPE
MDHCNet-DWI	0.790 ± 0.027	0.733 ± 0.144	0.626 ± 0.080	0.856 ± 0.037
MDHCNet-MRA	0.795 ± 0.055	0.762 ± 0.099	0.701 ± 0.055	0.834 ± 0.088
MDHCNet-T1W	0.740 ± 0.040	0.700 ± 0.095	0.676 ± 0.137	0.767 ± 0.071
MDHCNet-T2W	0.777 ± 0.015	0.749 ± 0.057	0.668 ± 0.164	0.825 ± 0.062
MDHCNet-all	**0.851** ± **0.039**	**0.795** ± **0.097**	**0.755** ± **0.167**	**0.893** ± **0.043**

### Comparison with related methods

To validate the effectiveness and superiority of MDHCNet, comparative experiments were conducted against established methods commonly employed for PSCI prediction. Specifically, the following approaches were included: (1) Clinical+SVM [6]: A conventional machine learning approach that utilizes clinical features with SVM classifier; (2) Radiomics+Logistic [7]: A radiomics-based method that extracts quantitative features from DWI sequences within manually delineated lesion regions, followed by feature selection using Spearman correlation analysis and mRMR algorithm, and logistic regression for final prediction; (3) MHGSA [13]: A SOTA multimodal approach that integrates clinical and imaging data through a dynamic GCN architecture for PSCI prediction.

The comparative results (Table 8) demonstrate that MDHCNet achieved superior performance across all evaluation metrics. The radiomics-based approach showed the poorest performance, with a low SEN (0.350 ± 0.163) and AUC (0.502 ± 0.040), indicating that radiomics features based on lesions are insufficient for PSCI prediction. While the MHGSA method demonstrated competitive performance, MDHCNet still outperformed it with improvements in ACC (0.851 ± 0.039 *vs* 0.801 ± 0.066), SEN (0.755 ± 0.167 *vs* 0.705 ± 0.221), and SPE (0.893 ± 0.043 *vs* 0.843 ± 0.077), validating the importance of HC and fusion strategies for multimodal integration.

**Table 8:** Comparison with related methods

MRI data	ACC	AUC	SEN	SPE
Clinical+SVM [6]	0.675 ± 0.052	0.700 ± 0.137	0.575 ± 0.209	0.719 ± 0.083
Radiomics+Logistic [7]	0.555 ± 0.077	0.502 ± 0.040	0.350 ± 0.163	0.633 ± 0.150
MHGSA [13]	0.801 ± 0.066	0.792 ± 0.068	0.705 ± 0.221	0.843 ± 0.077
MDHCNet	**0.851** ± **0.039**	**0.795** ± **0.097**	**0.755** ± **0.167**	**0.893** ± **0.043**

### Visualization and analysis of network representations

To enhance interpretability, this study visualizes the attention weights from the node enhancement module ($A_{\text {node}}$), edge enhancement weights ($A_{\text {edge}}$), and the hierarchical clustering process.

**Fig. 4:**
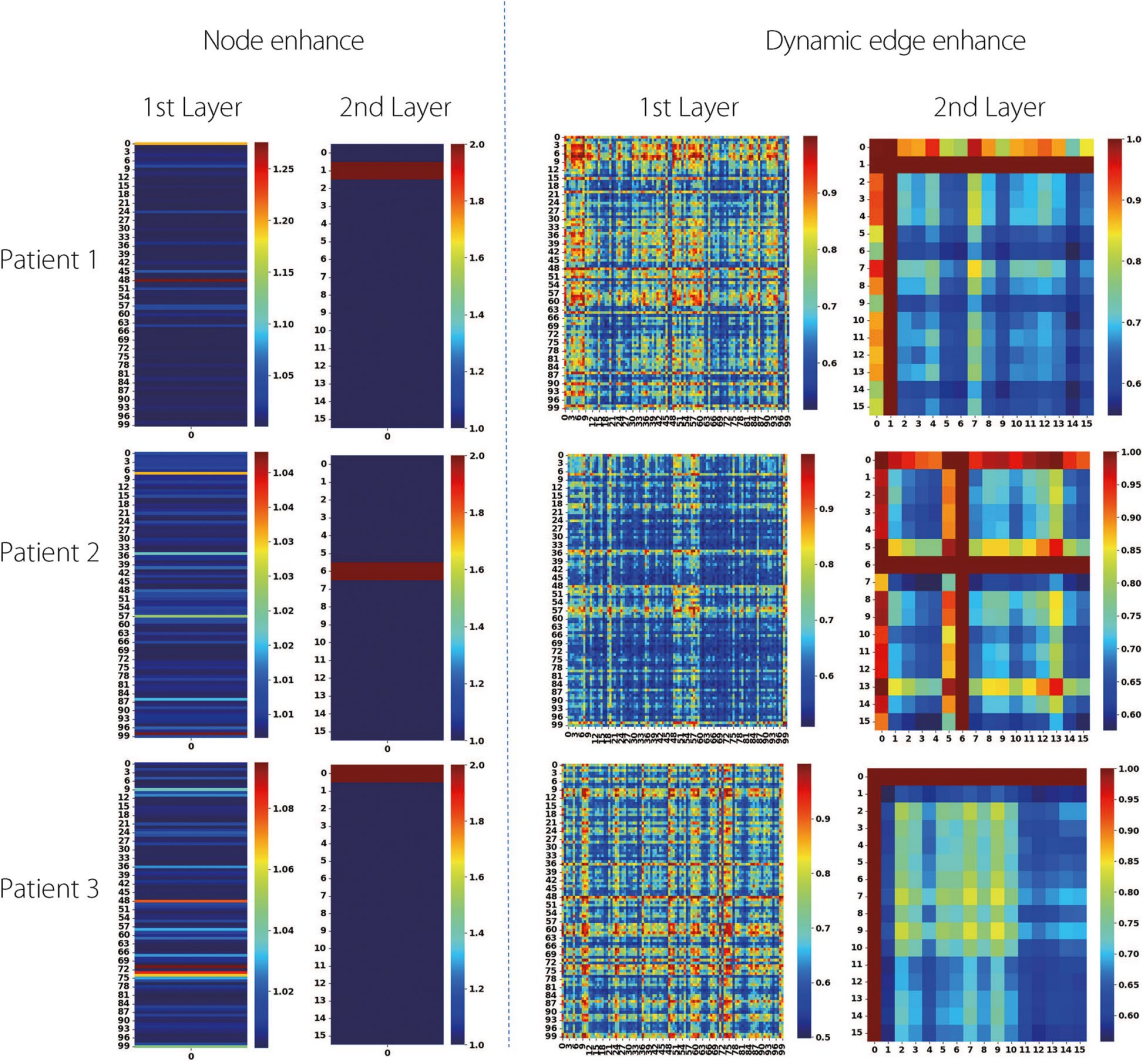
Visualization of node and edge enhancements. In the node enhancement map, darker colors indicate a higher contribution of a node to the model’s prediction. In the edge enhancement map, darker edges represent stronger learned connectivity between corresponding brain regions. Patient 1 represents a non-PSCI case, while Patients 2 and 3 are PSCI cases

**Fig. 5:**
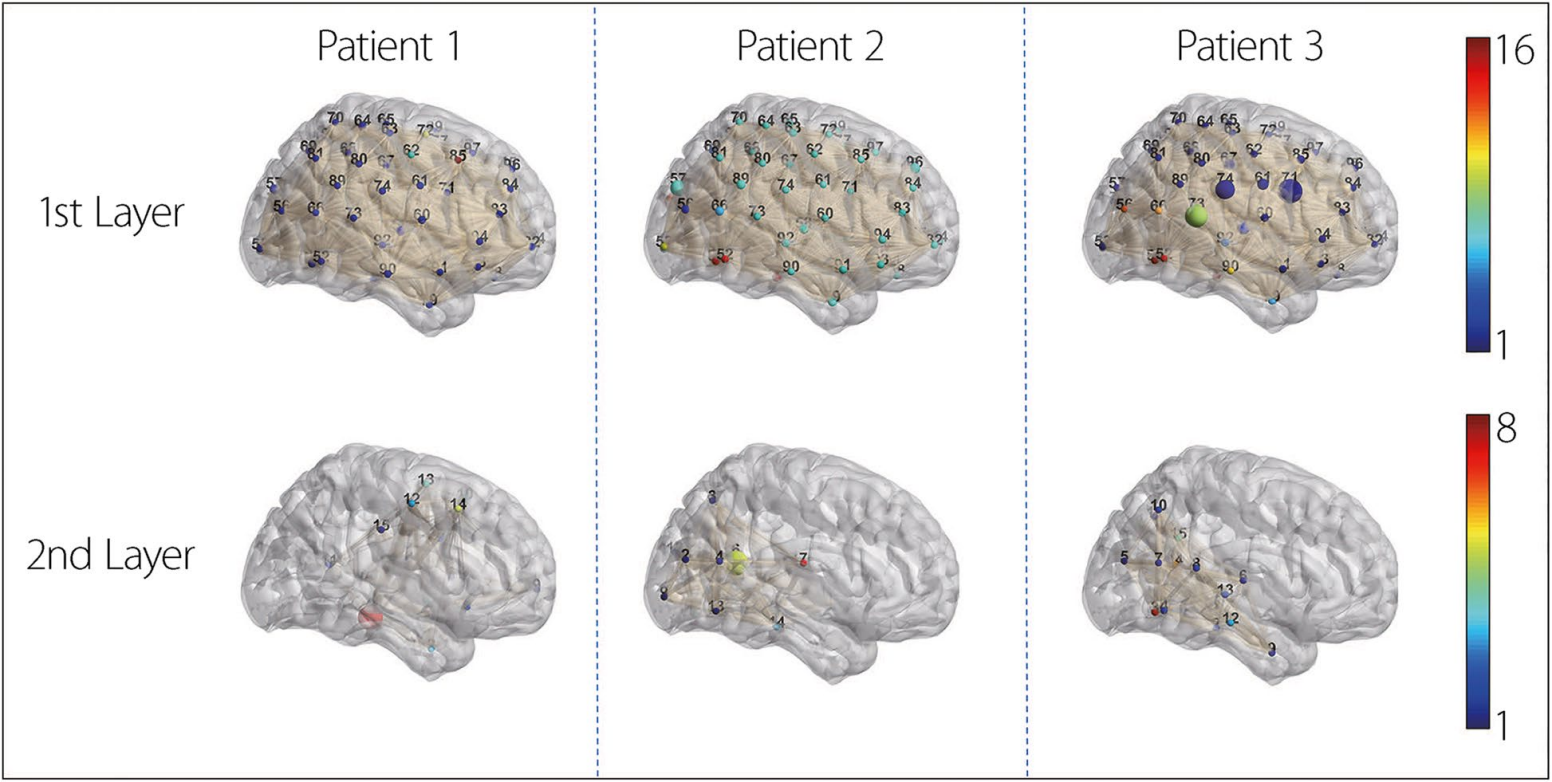
3D visualization of HC results. In the first layer, nodes represent individual brain regions; in the second layer, nodes denote clusters obtained via hierarchical grouping. Larger node size indicates greater predictive contribution. Node color denotes cluster identity, while edge thickness reflects connection strength, with thicker edges indicating stronger inter-regional relationships. Patient 1 represents a non-PSCI case, while Patients 2 and 3 are PSCI cases

**Node and edge attention.** Figure 4 illustrates the attention scores of 100 nodes (detailed in Table S1) and 16 clusters (detailed in Tables S2-S4) in a two-layer GCN. The color intensity represents the node importance, with red indicating a higher contribution. For three patients, the most salient regions in the first layer were node 48 (left hemisphere Precuneus Posterior Cingulate Cortex), 98 (right hemisphere Precuneus Posterior Cingulate Cortex), and 71 (right hemisphere Precentral Ventra); in the second layer, the most prominent clusters were cluster 1 (Visual), 6 (Precuneus Posterior Cingulate Cortex), and 0 (Precuneus Posterior Cingulate Cortex). Although PSCI and non-PSCI patients may share similar high-attention nodes in the first layer, this reflects only early-stage, shallow feature extraction. The underlying connectivity patterns differed substantially, as shown in the second layer. Specifically, PSCI patients (Patients 2 and 3) exhibited dominant attention toward Default Network-related clusters (clusters 6 and 0), whereas the non-PSCI patient (Patient 1) showed a stronger reliance on the visual cluster (cluster 1). Dynamic edge visualizations revealed that high-contribution nodes had stronger edge connections with other nodes. For instance, in the first layer, node 48 of Patient 1 exhibited the highest attention and the strongest connectivity to other nodes. A similar pattern was observed in the second layer of cluster 1. This suggests that high-importance nodes not only contribute significantly to classification but also play key roles in altering the brain’s connectivity structure.

**HC visualization.** Figure 5 presents a three-dimensional view of the clustering process. In the first layer, 100 brain regions were grouped into 16 clusters; in the second layer, these were further merged into 8 meta-clusters. Node size indicates importance, and color denotes cluster membership. The edges reflect the connection strength, with a thickness proportional to the edge weight. To map the cluster locations back to their anatomy, each cluster was mapped based on the most important node it contained. The PSCI patients (Patients 2 and 3) exhibited the highest attention weights in clusters associated with the Default Network (clusters 6 and 0, corresponding to the Precuneus and Posterior Cingulate Cortex). This suggests that cognitive impairment in PSCI is primarily driven by disruptions within the Default Network, which is consistent with its known role in attention regulation and memory consolidation. In contrast, the non-PSCI patients without PSCI primarily relied on visual processing clusters, indicating a relatively preserved higher-order cognitive network function. These results demonstrate the model’s ability to capture meaningful network-level patterns associated with PSCI and highlight the relevance of hierarchical brain organization in understanding its pathophysiology.

## Discussion

This section analyzes the experimental findings from multiple perspectives, including contributions of the key module, generalization ability, current limitations, and potential improvements to the proposed method.

**Importance of model components.** Ablation studies demonstrated the critical role played by each module in the overall performance. The clinical data-guided node enhancement module significantly improved node representation, particularly for low-signal nodes. The dynamic edge enhancement module, consisting of learnable edge weights and brain network damage information, facilitated the flexible modeling of both functional and structural connectivity. The removal of either component led to performance degradation, confirming their synergistic benefits.

**Benefits of the HC module.** The HC module plays a dual role: improving the classification ACC and enhancing interpretability. It adaptively identifies important substructures within the brain network by grouping nodes based on their similarity. The experimental results show that performance deteriorated when clustering was removed or its associated losses were ablated. Furthermore, the visualization results revealed that cluster assignments aligned with known anatomical regions, providing clinical insight into PSCI-related network reorganization. Therefore, the HC module acts as both a structural regularizer and visual explainer.

**Generalization ability.** MDHCNet achieved SOTA performance, indicating strong generalization capability. The inclusion of modality-guided attention mechanisms and a compact hierarchical structure likely contributed to its robustness and adaptability.

**Limitations and future work.** Despite its promising performance, MDHCNet has several limitations. First, current clustering is performed independently on each graph, which may limit cross-subject consistency. Future research will explore group-wise and population-level clustering strategies. Second, the current framework focuses on classification. Future extensions could include longitudinal modeling to predict cognitive decline trajectories. In addition, although the proposed method enhances interpretability, further efforts are required to validate the biological relevance of the identified clusters via neuroscientific or clinical correlates.

In summary, MDHCNet demonstrates the feasibility and effectiveness of integrating multimodal brain imaging and clinical data for PSCI diagnosis while offering interpretable insights through hierarchical graph modeling. This study provides a practical foundation for future research on neurodegenerative disease diagnosis based on graph learning paradigms.

## Conclusions

This study proposed MDHCNet, a novel multimodal brain network GCN for PSCI classification. By integrating DWI, MRA, T1W, T2W, brain network damage matrix and clinical data, the model effectively captured heterogeneous pathological characteristics of PSCI. The decoupled hierarchical fusion framework enables both intra- and intermodal feature interactions, whereas the integration of clinical guidance and dynamic edge modeling enhances node and connectivity representations. Furthermore, the HC module not only improves classification performance but also enhances the interpretability of the learned brain network substructures. The experimental results demonstrate the superiority and generalizability of MDHCNet over existing baselines. Importantly, the proposed method provides clinically meaningful insights into the network reorganization patterns associated with cognitive impairment after stroke. Future work will focus on extending the framework to longitudinal modeling and improving the clinical relevance of the discovered biomarkers.

## Supplementary information

Supplementary Material 1.

## Data Availability

Clinical data are not publicly available as they contain private patient health information. To ensure ethical compliance, approval was obtained from the local medical ethics committee. The requirement for informed consent was waived.

## Abbreviations

ACC: Accuracy
AUC: Area under the ROC curve
DWI: Diffusion-weighted imaging
GCN: Graph convolutional network
GLCM: Gray-level co-occurrence matrix
GLDM: Gray-level dependence matrix
GLRLM: Gray-level run-length matrix
GLSZM: Gray-level size zone matrix
H: High-pass filter
HC: Hierarchical clustering
L: Low-pass filter
MDHCNet: Multimodal dynamic hierarchical clustering network
MoCA: Montreal cognitive assessment
MRA: Magnetic resonance angiography
MRI: Magnetic resonance imaging
mRMR: Minimum redundancy maximum relevance
NGTDM: Neighboring gray-tone difference matrix
NIHSS: National institutes of health stroke scale
PSCI: Post-stroke cognitive impairment
SD: Structural damage
SEN: Sensitivity
SOTA: State-of-the-art
SPE: Specificity
SVM: Support vector machine
T1W: T1-weighted image
T2W: T2-weighted image
