# Supplementary material for: Multimodal dynamic hierarchical clustering model for post-stroke cognitive impairment prediction
Source: Vis Comput Ind Biomed Art. 2025 Sep 1;8:20. doi: 10.1186/s42492-025-00202-0 (PMC12401840; doi:10.1186/s42492-025-00202-0)
Supplement: Supplementary file 1 — Supplementary Material 1. [file 42492_2025_202_MOESM1_ESM.docx]

Table S1: Definitions and descriptions of the 100 brain regions in the first layer of model Visualization (LH, left hemisphere; RH,right hemisphere)

| **NO.** | **Network** | **Brain Parcels** | **Network Function** |
| --- | --- | --- | --- |
| **0** | **Visual Network** | **LH_Visual_1** | The Visual Network is responsible for processing visual information. It primarily includes the primary and secondary visual cortices (V1, V2, V3, V4), and is involved in the perception of light, shapes, and motion. It plays a crucial role in visual perception, recognition, and spatial orientation. |
| **1** |  | **LH_Visual_2** |  |
| **2** |  | **LH_Visual_3** |  |
| **3** |  | **LH_Visual_4** |  |
| **4** |  | **LH_Visual_5** |  |
| **5** |  | **LH_Visual_6** |  |
| **6** |  | **LH_Visual_7** |  |
| **7** |  | **LH_Visual_8** |  |
| **8** |  | **LH_Visual_9** |  |
| **9** | **Somatomotor Network** | **LH_Somatomotor_1** | Key areas of this network include the primary motor cortex and somatosensory cortex, which coordinate movement and the perception of bodily sensations. |
| **10** |  | **LH_Somatomotor_2** |  |
| **11** |  | **LH_Somatomotor_3** |  |
| **12** |  | **LH_Somatomotor_4** |  |
| **13** |  | **LH_Somatomotor_5** |  |
| **14** |  | **LH_Somatomotor_6** |  |
| **15** | **Dorsal Attention Network** | **LH_Dorsal Attention_Post_1** | It is involved in voluntary (top-down) orienting and shows activity increases after presentation of cues indicating where, when, or to what subjects should direct their attention. |
| **16** |  | **LH_Dorsal Attention_Post_2** |  |
| **17** |  | **LH_Dorsal Attention_Post_3** |  |
| **18** |  | **LH_Dorsal Attention_Post_4** |  |
| **19** |  | **LH_Dorsal Attention_Post_5** |  |
| **20** |  | **LH_Dorsal Attention_Post_6** |  |
| **21** |  | **LH_Dorsal Attention_Precentral Ventral_1** |  |
| **22** |  | **LH_Dorsal Attention_Frontal Eye Fields_1** |  |
| **23** | **Salience/ Ventral Attention Network** | **LH_Salience/ Ventral Attention_ Parietal Operculum_1** | Salience Network: Detection of important stimuli and emotional regulation. Ventral Attention Network: It shows activity increases upon detection of salient targets, especially when they appear in unexpected locations. |
| **24** |  | **LH_Salience/ Ventral Attention_ Frontal Operculum Insula_1** |  |
| **25** |  | **LH_Salience/ Ventral Attention_Frontal Operculum Insula_2** |  |
| **26** |  | **LH_Salience/ Ventral Attention_ Lateral Prefrontal Cortex_1** |  |
| **27** |  | **LH_Salience/ Ventral Attention_ Medial_1** |  |
| **28** |  | **LH_Salience/ Ventral Attention_ Medial_2** |  |
| **29** |  | **LH_Salience/ Ventral Attention_ Medial_3** |  |
| **30** | **Limbic Network** | **LH_Limbic_Orbital Frontal Cortex_1** | The Limbic Network connects emotion, memory, and motivation, providing the foundation for emotional regulation, social behavior, and adaptive responses to internal and external environments. |
| **31** |  | **LH_Limbic_Temporal Pole_1** |  |
| **32** |  | **LH_Limbic_ Temporal Pole_2** |  |
| **33** | **Control Network** | **LH_Control_ Parietal_1** | Executive control and task-switching. |
| **34** |  | **LH_Control_ Lateral Prefrontal Cortex_1** |  |
| **35** |  | **LH_Control_Precuneus_1** |  |
| **36** |  | **LH_Control_Cingulate_1** |  |
| **37** | **Default network** | **LH_Default_ Temporal_1** | The network is closely involved in episodic memory processing. |
| **38** |  | **LH_Default_Temporal_2** |  |
| **39** |  | **LH_Default_ Parietal_1** |  |
| **40** |  | **LH_Default_ Parietal_2** |  |
| **41** |  | **LH_Default_Prefrontal Cortex_1** |  |
| **42** |  | **LH_Default_Prefrontal Cortex_2** |  |
| **43** |  | **LH_Default_Prefrontal Cortex_3** |  |
| **44** |  | **LH_Default_Prefrontal Cortex_4** |  |
| **45** |  | **LH_Default_Prefrontal Cortex_5** |  |
| **46** |  | **LH_Default_Prefrontal Cortex_6** |  |
| **47** |  | **LH_Default_Prefrontal Cortex_7** |  |
| **48** |  | **LH_Default_ Precuneus Posterior Cingulate Cortex_1** |  |
| **49** |  | **LH_Default_Precuneus Posterior Cingulate Cortex_2** |  |
| **50** | **Visual Network** | **RH_Visual_1** | The Visual Network is responsible for processing visual information. It primarily includes the primary and secondary visual cortices (V1, V2, V3, V4), and is involved in the perception of light, shapes, and motion. It plays a crucial role in visual perception, recognition, and spatial orientation. |
| **51** |  | **RH_Visual_2** |  |
| **52** |  | **RH_Visual_3** |  |
| **53** |  | **RH_Visual_4** |  |
| **54** |  | **RH_Visual_5** |  |
| **55** |  | **RH_Visual_6** |  |
| **56** |  | **RH_Visual_7** |  |
| **57** |  | **RH_Visual_8** |  |
| **58** | **Somatomotor Network** | **RH_Somatomotor_1** | Key areas of this network include the primary motor cortex and somatosensory cortex, which coordinate movement and the perception of bodily sensations. |
| **59** |  | **RH_Somatomotor_2** |  |
| **60** |  | **RH_Somatomotor_3** |  |
| **61** |  | **RH_Somatomotor_4** |  |
| **62** |  | **RH_Somatomotor_5** |  |
| **63** |  | **RH_Somatomotor_6** |  |
| **64** |  | **RH_Somatomotor_7** |  |
| **65** |  | **RH_Somatomotor_8** |  |
| **66** | **Dorsal Attention Network** | **RH_Dorsal Attention_Post_1** | It is involved in voluntary (top-down) orienting and shows activity increases after the presentation of cues indicating where, when, or to what subjects should direct their attention. |
| **67** |  | **RH_Dorsal Attention_Post_2** |  |
| **68** |  | **RH_Dorsal Attention_Post_3** |  |
| **69** |  | **RH_Dorsal Attention_Post_4** |  |
| **70** |  | **RH_Dorsal Attention_Post_5** |  |
| **71** |  | **RH_Dorsal Attention_ Precentral Ventra_1** |  |
| **72** |  | **RH_Dorsal Attention_ Frontal Eye Fields_1** |  |
| **73** | **Salience/ Ventral Attention Network** | **RH_Salience/ Ventral Attention_TempOccPar_1** | Salience Network: Detection of important stimuli and emotional regulation. Ventral Attention Network: It shows activity increases upon detection of salient targets, especially when they appear in unexpected locations. |
| **74** |  | **RH_Salience/ Ventral Attention_TempOccPar_2** |  |
| **75** |  | **RH_Salience/ Ventral Attention_Frontal Operculum Insula_1** |  |
| **76** |  | **RH_Salience/ Ventral Attention_Medial_1** |  |
| **77** |  | **RH_Salience/ Ventral Attention_Medial_2** |  |
| **78** | **Limbic Network** | **RH_Limbic_Orbital Frontal Cortex_1** | The Limbic Network connects emotion, memory, and motivation, providing the foundation for emotional regulation, social behavior, and adaptive responses to internal and external environments. |
| **79** |  | **RH_Limbic_Temporal Pole_1** |  |
| **80** | **Control Network** | **RH_Control_Parietal_1** | Executive control and task-switching. |
| **81** |  | **RH_Control_Parietal_2** |  |
| **82** |  | **RH_Control_Lateral Prefrontal Cortex_1** |  |
| **83** |  | **RH_Control_Lateral Prefrontal Cortex_2** |  |
| **84** |  | **RH_Control_Lateral Prefrontal Cortex_3** |  |
| **85** |  | **RH_Control_Lateral Prefrontal Cortex_4** |  |
| **86** |  | **RH_Control_Cingulate_1** |  |
| **87** |  | **RH_Control_medial posterior Prefrontal Cortex_1** |  |
| **88** |  | **RH_Control_Precuneus_1** |  |
| **89** | **Default Network** | **RH_Default_Parietal_1** | The network is closely involved in episodic memory processing. |
| **90** |  | **RH_Default_Temporal_1** |  |
| **91** |  | **RH_Default_Temporal_2** |  |
| **92** |  | **RH_Default_Temporal_3** |  |
| **93** |  | **RH_Default_ventrolateral posterior Prefrontal Cortex_1** |  |
| **94** |  | **RH_Default_ventrolateral posterior Prefrontal Cortex_2** |  |
| **95** |  | **RH_Default_Prefrontal Cortex dorsal and Prefrontal Cortex medial_1** |  |
| **96** |  | **RH_Default_Prefrontal Cortex dorsal and Prefrontal Cortex medial_2** |  |
| **97** |  | **RH_Default_Prefrontal Cortex dorsal and Prefrontal Cortex medial_3** |  |
| **98** |  | **RH_Default_Precuneus Posterior Cingulate Cortex_1** |  |
| **99** |  | **RH_Default_ Precuneus Posterior Cingulate Cortex_2** |  |

Table S2: Representative brain regions of 16 clusters in the second layer of **Patient 1**'s visualization model (using the highest-contributing brain region node as the cluster representative)

| Cluster | Brain region |
| --- | --- |
| 0 | LH_Salience/ Ventral Attention_ Frontal Operculum Insula_1 |
| 1 | LH_Visual_1 |
| 2 | LH_Limbic_ Temporal Pole_1 |
| 3 | LH_Somatomotor_2 |
| 4 | LH_Somatomotor_3 |
| 5 | LH_Dorsal Attention_Precentral Ventral_1 |
| 6 | LH_Salience/ Ventral Attention_Frontal Operculum Insula_2 |
| 7 | LH_Limbic_ Temporal Pole_2 |
| 8 | LH_Default_Prefrontal Cortex_1 |
| 9 | LH_Default_Prefrontal Cortex_4 |
| 10 | LH_Default_Prefrontal Cortex_7 |
| 11 | LH_Default_ Precuneus Posterior Cingulate Cortex_1 |
| 12 | RH_Somatomotor_5 |
| 13 | RH_Dorsal Attention_ Frontal Eye Fields_1 |
| 14 | RH_Control_Lateral Prefrontal Cortex_4 |
| 15 | RH_Control_Cingulate_1 |

Table S3: Representative brain regions of 16 clusters in the second layer of **Patient 2**'s visualization model (using the highest-contributing brain region node as the cluster representative)

| Cluster | Brain region |
| --- | --- |
| 0 | LH_Salience/ Ventral Attention_ Frontal Operculum Insula_1 |
| 1 | LH_Somatomotor_2 |
| 2 | RH_Visual_7 |
| 3 | RH_Dorsal Attention_Post_4 |
| 4 | RH_Dorsal Attention_Post_1 |
| 5 | LH_Visual_1 |
| 6 | RH_Default_Precuneus Posterior Cingulate Cortex_1 |
| 7 | RH_Somatomotor_2 |
| 8 | LH_Dorsal Attention_Post_3 |
| 9 | RH_Visual_4 |
| 10 | LH_Default_ Parietal_2 |
| 11 | LH_Visual_7 |
| 12 | LH_Visual_8 |
| 13 | RH_Visual_2 |
| 14 | RH_Visual_1 |
| 15 | LH_Visual_2 |

Table S4: Representative brain regions of 16 clusters in the second layer of **Patient 3**'s visualization model (using the highest-contributing brain region node as the cluster representative)

| Cluster | Brain region |
| --- | --- |
| 0 | LH_Default_ Precuneus Posterior Cingulate Cortex_1 |
| 1 | LH_Control_Precuneus_1 |
| 2 | RH_Visual_1 |
| 3 | RH_Visual_2 |
| 4 | RH_Visual_3 |
| 5 | RH_Visual_7 |
| 6 | RH_Somatomotor_1 |
| 7 | RH_Dorsal Attention_Post_1 |
| 8 | RH_Salience/ Ventral Attention_Temporal-Occipital-Parietal_1 |
| 9 | RH_Limbic_Temporal Pole_1 |
| 10 | RH_Control_Parietal_2 |
| 11 | RH_Control_Precuneus_1 |
| 12 | RH_Default_Temporal_1 |
| 13 | RH_Default_Temporal_3 |
| 14 | RH_Default_Precuneus Posterior Cingulate Cortex_1 |
| 15 | RH_Default_ Precuneus Posterior Cingulate Cortex_2 |
